# COVID-19 and pneumothorax: a multicentre retrospective case series

**DOI:** 10.1183/13993003.02697-2020

**Published:** 2020-11-19

**Authors:** Anthony W. Martinelli, Tejas Ingle, Joseph Newman, Iftikhar Nadeem, Karl Jackson, Nicholas D. Lane, James Melhorn, Helen E. Davies, Anthony J. Rostron, Aldrin Adeni, Kevin Conroy, Nick Woznitza, Matthew Matson, Simon E. Brill, James Murray, Amar Shah, Revati Naran, Samanjit S. Hare, Oliver Collas, Sarah Bigham, Michael Spiro, Margaret M. Huang, Beenish Iqbal, Sarah Trenfield, Stephane Ledot, Sujal Desai, Lewis Standing, Judith Babar, Razeen Mahroof, Ian Smith, Kai Lee, Nairi Tchrakian, Stephanie Uys, William Ricketts, Anant R.C. Patel, Avinash Aujayeb, Maria Kokosi, Alexander J.K. Wilkinson, Stefan J. Marciniak

**Affiliations:** 1 Addenbrooke's Hospital, Cambridge, UK; 2 CITIID, University of Cambridge, Cambridge, UK; 3 The Lister Hospital, Stevenage, UK; 4 Bedford Hospital, Bedford, UK; 5 Northumbria Specialist Emergency Care Hospital, Cramlington, UK; 6 Respiratory Medicine, The Royal Victoria Infirmary, Newcastle-upon-Tyne, UK; 7 Translational and Clinical Research Institute, Newcastle University, Newcastle upon Tyne, UK; 8 University Hospital of Wales, Cardiff, UK; 9 South Tyneside and Sunderland NHS Foundation Trust, Sunderland, UK; 10 Doncaster and Bassetlaw Teaching Hospitals NHS Foundation Trust, Doncaster, UK; 11 University Hospital of North Tees, Stockton, UK; 12 NHS Nightingale Hospital, Royal Docks, London, UK; 13 Canterbury Christ Church University, Canterbury, UK; 14 Royal Free London NHS Foundation Trust, Hampstead, London, UK; 15 Royal Papworth Hospital, Cambridge, UK; 16 King's College Hospital, London, UK; 17 Royal Brompton Hospital, London, UK; 18 Queen Elizabeth Hospital, Gateshead, UK; 19 The Royal London Hospital, London, UK; 20 CIMR, University of Cambridge, Cambridge, UK; 21 Both authors contributed equally

## Abstract

**Introduction:**

Pneumothorax and pneumomediastinum have both been noted to complicate cases of coronavirus disease 2019 (COVID-19) requiring hospital admission. We report the largest case series yet described of patients with both these pathologies (including nonventilated patients).

**Methods:**

Cases were collected retrospectively from UK hospitals with inclusion criteria limited to a diagnosis of COVID-19 and the presence of either pneumothorax or pneumomediastinum. Patients included in the study presented between March and June 2020. Details obtained from the medical record included demographics, radiology, laboratory investigations, clinical management and survival.

**Results:**

71 patients from 16 centres were included in the study, of whom 60 had pneumothoraces (six with pneumomediastinum in addition) and 11 had pneumomediastinum alone. Two of these patients had two distinct episodes of pneumothorax, occurring bilaterally in sequential fashion, bringing the total number of pneumothoraces included to 62. Clinical scenarios included patients who had presented to hospital with pneumothorax, patients who had developed pneumothorax or pneumomediastinum during their inpatient admission with COVID-19 and patients who developed their complication while intubated and ventilated, either with or without concurrent extracorporeal membrane oxygenation. Survival at 28 days was not significantly different following pneumothorax (63.1±6.5%) or isolated pneumomediastinum (53.0±18.7%; p=0.854). The incidence of pneumothorax was higher in males. 28-day survival was not different between the sexes (males 62.5±7.7% *versus* females 68.4±10.7%; p=0.619). Patients aged ≥70 years had a significantly lower 28-day survival than younger individuals (≥70 years 41.7±13.5% survival *versus* <70 years 70.9±6.8% survival; p=0.018 log-rank).

**Conclusion:**

These cases suggest that pneumothorax is a complication of COVID-19. Pneumothorax does not seem to be an independent marker of poor prognosis and we encourage continuation of active treatment where clinically possible.

## Abstract 2

**Roughly 1% of patients admitted with COVID-19 develop pneumothorax. This can occur without pre-existing lung disease or mechanical ventilation. Two-thirds of patients survive, but age >70 years and acidosis are associated with poor prognosis.** https://bit.ly/2Z1mfeo

## Introduction

Pneumothorax has been reported in a small number of patients with coronavirus disease 2019 (COVID-19), although the significance and frequency of this association remain unclear. Retrospective studies of patients with COVID-19 have suggested that pneumothorax might occur in 1% of those requiring hospital admission, 2% of patients requiring intensive care unit (ICU) admission and 1% of patients dying from the infection [1–3]. More recently, the rate of barotrauma, comprising both pneumothorax and pneumomediastinums in ventilated patients has been reported as 15% [4]. The current literature consists primarily of single case reports, with the largest published series comprising three patients who died with COVID-19 and pneumomediastinum, two of whom also had pneumothorax [5]. Pneumomediastinum has also been described as a complication of COVID-19, both in patients breathing spontaneously and as a consequence of invasive positive-pressure ventilation [6, 7].

Spontaneous pneumothorax was reported as a complication of severe acute respiratory syndrome (SARS, caused by SARS-coronavirus (CoV)-1) with an incidence of 1.7% in hospitalised patients [8]. In that retrospective case series of six patients, four were admitted to the ICU and two died. Pneumothorax was more likely in patients with neutrophilia, severe lung injury and a protracted clinical course. Similarly, pneumothorax was noted as a poor prognostic feature of Middle East respiratory syndrome-related coronavirus infection [9].

Here, we report the largest case series to date of pneumothorax with COVID-19 to include nonintubated patients, revealing that it occurs even in patients with no pre-existing lung disease who have not required positive-pressure ventilation. Our study aims to describe the clinical characteristics of patients with these pathologies and consider whether development of pneumothorax can be used as a marker of poor prognosis.

## Methods

### Patients

Ethical approval was obtained from the Cambridge University Hospitals NHS Foundation Trust (UK) audit committee with additional Caldicott approval from local trusts where relevant. Cases were collected retrospectively based on a combination of author recall and targeted review of hospital coding databases from across the UK. The initial appeal for cases was among East of England respiratory trainees, which was then expanded nationally *via* a call for collaboration on Twitter. Inclusion criteria were limited to a diagnosis of COVID-19 and the presence of either pneumothorax or pneumomediastinum, with patients presenting between March and the beginning of June 2020, allowing for ≥28 days of follow-up post-pneumothorax in each case. Details obtained from the medical record included demographics (ages were limited to bands of one decade to maintain patient anonymity), past medical history, laboratory investigations (including full blood count, C-reactive protein and D-dimer), radiological findings (chest radiograph and computed tomography (CT)), clinical management, patient progress and survival (tables 1 and 2 and supplementary tables S1 and S2).

**TABLE 1:** Demographic and clinical details for pneumothorax and coronavirus disease 2019

**Age years**	
21–30	1 (2)
31–40	4 (7)
41–50	10 (17)
51–60	16 (27)
61–70	14 (23)
71–80	15 (25)
**Female**	14 (23)
**Side affected**	
Right	38 (63)
Left	18 (30)
Bilateral	4 (7)
**Smoking status**	
Never-smoker	34 (57)
Current smoker	3 (5)
Ex-smoker	15 (25)
Unknown	8 (13)
**Respiratory comorbidities**	
COPD	6 (10)
Asthma	10 (17)
Bronchiectasis	2 (3)
Pneumothorax	0 (0)
None	43 (72)
**Other comorbidities**	
Systemic hypertension	19 (32)
Hyperlipidaemia	16 (27)
Atrial fibrillation	3 (5)
Chronic kidney disease	4 (7)
Type 2 diabetes mellitus	10 (17)
**Mode of breathing^#^**	
Spontaneous	20 (32)
CPAP/NIV	3 (5)
Intubated and ventilated	27 (44)
ECMO	12 (20)
**Mode of diagnosis^#^**	
On admission	9 (15)
Clinical change	17 (27)
Incidental	31 (50)
Clinical examination	3 (5)
Unknown	2 (3)
**Management^#^**	
Chest drain	43 (69)
Chest drain then surgery	1 (2)
Conservative	15 (24)
Palliative	3 (5)
**Height^¶^ m**	1.72 (1.67–1.79)
**BMI^+^ kg·m^−2^**	27.0 (23.5–31.4)

Data are presented as n (%) or median (interquartile range). CPAP: continuous positive airway pressure; NIV: noninvasive ventilation; ECMO: extracorporeal membrane oxygenation; BMI: body mass index. ^#^: n=62 due to two patients with sequential pneumothoraces; ^¶^: n=52; ^+^: n=53.

**TABLE 2:** Demographic and clinical details for pneumomediastinum and coronavirus disease 2019

**Age years**	
41–50	3 (27)
51–60	2 (18)
61–70	6 (55)
**Female**	0 (0)
**Mode of breathing**	
Spontaneous	1 (9)
CPAP/NIV	1 (9)
Intubated and ventilated	6 (55)
ECMO	3 (27)

Data are presented as n (%). CPAP: continuous positive airway pressure; NIV: noninvasive ventilation; ECMO: extracorporeal membrane oxygenation.

### Data and statistical analyses

Patient data are presented as absolute values, percentages, mean±se or median (interquartile rage (IQR)). Survival data were used to generate Kaplan–Meier curves using SPSS 26 (SPSS, Chicago, IL, USA). Survival was compared using the log-rank test; survival tables provided cumulative survival and standard error; life tables provided number exposed to risk at each timepoint. p-values <0.05 were considered statistically significant.

## Results

A total of 71 patients were reviewed, of whom 60 patients had pneumothoraces (six with pneumomediastinum in addition) and 11 had pneumomediastinum alone. Two patients had two distinct episodes of pneumothorax, occurring bilaterally in sequential fashion, bringing the total number of pneumothoraces included to 62. Details for the 60 patients with pneumothoraces are described in table 1. Details for the 11 cases of pneumomediastinum alone are shown in table 2. None of these patients required specific intervention for pneumomediastinum. Owing to their scarcity, patients with pneumomediastinum alone were not studied in further depth.

**FIGURE 1:**
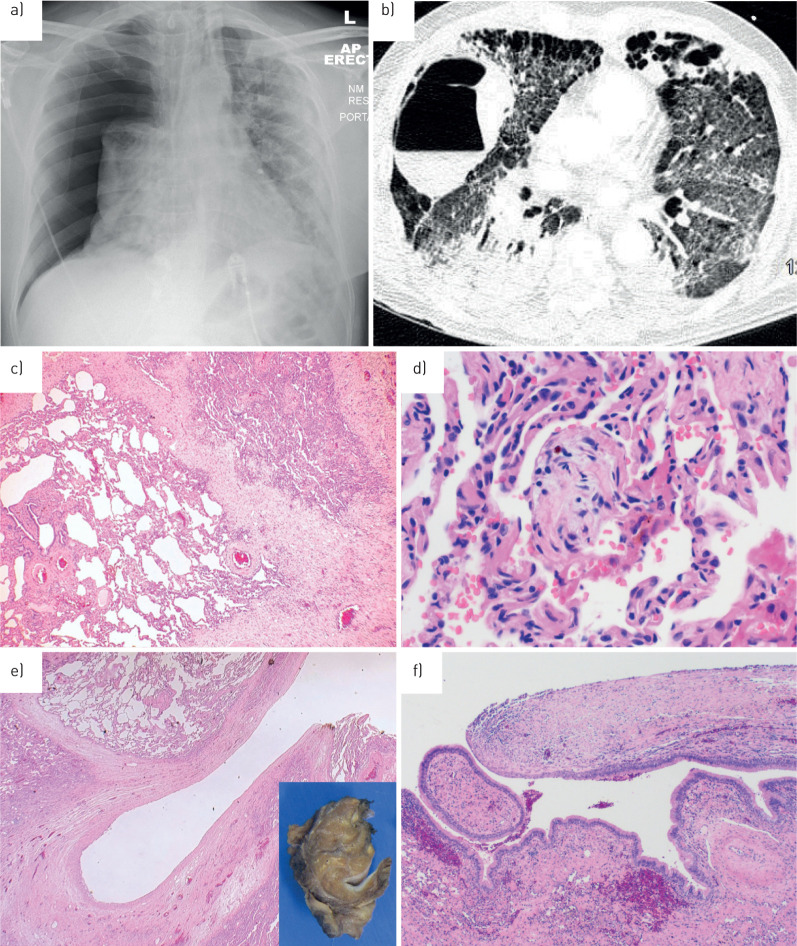
Radiology and pathology in pneumothorax coronavirus disease 2019 (COVID-19). a) Anteroposterior erect chest radiograph: a male is his sixties presenting with a large right pneumothorax and some leftward tracheal shift. Background widespread bilateral alveolar opacity is consistent with “classic” COVID. b) Axial computed tomography image of the thorax acquired in a COVID-19 patient shortly before development of a right-sided pneumothorax. Note a large right-sided thin-walled cavity with air–fluid level, as well as numerous subpleural cystic spaces in the anterior hemithoraces bilaterally. c) Medium-power photomicrograph of lung parenchyma showing foci of collapse with accompanying fibrosis and vascular congestion. d) High-power image of intra-alveolar fibromyxoid plugs, fibrin and haemosiderin deposition. e) Low-power view of the 15-mm cystic space with a thick, fibrotic wall (inset: corresponding macroscopic cross-section). f) Medium-power image of the fibrous cyst wall (right) transitioning with respiratory epithelium (left), suggesting possible connection with the bronchial tree.

Of the 60 patients with pneumothorax, 58 were laboratory-confirmed COVID-19 infection with two diagnosed based on clinical history and radiology. 61 pneumothoraces were confirmed by chest radiograph, while one case was diagnosed by suspicious radiology prior to deterioration. Typically, plain chest films provided no additional cause for the pneumothorax apart from concomitant COVID-19 (figure 1a). During the course of their admission, 37 patients underwent further cross-sectional imaging with CT scanning of the thorax. In one instance, CT suggested that pulmonary infarction might have triggered parenchymal cavitation with subsequent pleural rupture causing pneumothorax (figure 1b).

Surgical pathology was available from one patient who underwent bullectomy. This showed nonspecific changes including localised collapse and fibrosis, vascular congestion, scattered mild chronic inflammation and features of possible reparative change including haemosiderin deposition, fibrin collections and occasional fibromyxoid plugs within airspaces (figure 1c and d). Additionally, a 15-mm cystic space was identified grossly within the parenchyma; histological sections showed a dense fibrous cyst wall lined by bland cuboidal cells (figure 1e). Focally, the lining of the cystic space transitioned with respiratory epithelium, supportive of the radiological impression of pneumatocoele formation (figure 1f). There was no evidence of hyaline membrane formation, intra-alveolar proteinaceous exudates or viral cytopathic change.

**FIGURE 2:**
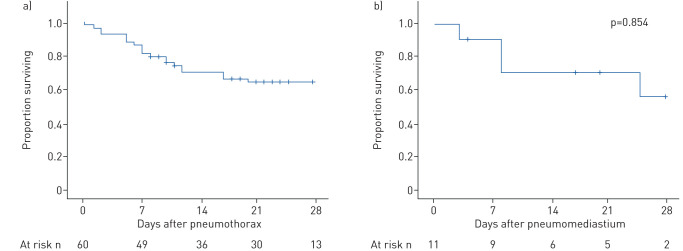
Mortality following pneumothorax or pneumomediastinum in coronavirus disease 2019. Kaplan–Meier survival curves from time of diagnosis of a) pneumothorax; b) pneumomediastinum. Patients at risk at each time point are indicated below each chart. Log-rank test comparing a) and b) p=0.854.

**FIGURE 3:**
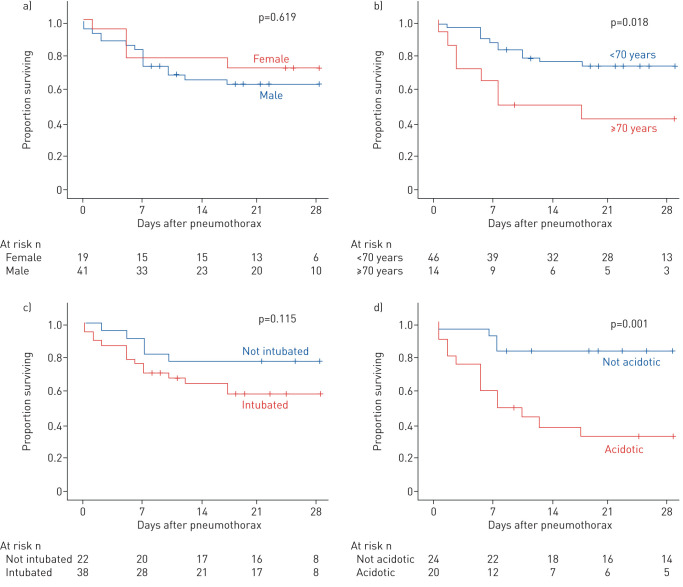
Relationship between sex, age, ventilatory support and pH and mortality in pneumothorax coronavirus disease 2019. Kaplan–Meier survival curves from time of diagnosis of pneumothorax: a) males or females log-rank test p=0.454; b) patients aged <70 years or ≥70 years log-rank test p=0.012; c) patients not intubated or receiving invasive ventilatory support with or without additional extracorporeal membrane oxygenation log-rank test p=0.173; d) patients with arterial pH ≥7.35 or <7.35 log-rank test p=0.001. Follow-up of 28 days, or to cross.

Overall survival at 28 days was not significantly different following pneumothorax (63.1±6.5%) or isolated pneumomediastinum (53.0±18.7%; p=0.854) (figure 2). The incidence of pneumothorax was higher in males. The 28-day survival was not different between the sexes (males 62.5±7.7% *versus* females 68.4±10.7%; p=0.619) (figure 3a). Patients aged ≥70 years had a significantly lower 28-day survival than younger individuals (≥70 years 41.7±13.5% survival *versus* <70 years 70.9±6.8% survival; p=0.018 log-rank) (figure 3b).

### Pneumothorax

Pneumothorax cases fell into three categories, as follows.

#### Acute presentation

Nine patients attended the emergency department with shortness of breath and were diagnosed with pneumothorax on initial chest radiograph; one patient showed signs of tension. Cough and chest pain were frequent symptoms, with tachypnoea and hypoxaemia common, but not universal. Five of these patients were readmissions to hospital: four having received recent inpatient treatment for COVID-19 and one thought to have contracted nosocomial COVID-19, which became evident on their second presentation.

Since all patients in this group were aged >40 years, they were atypical for primary spontaneous pneumothorax. Only two had existing pulmonary disease. It is therefore likely that pneumothorax in this group was a complication of COVID-19 rather than there being a chance association of the two conditions. Seven patients required intercostal chest drain insertion while two were managed conservatively. Two (22%) patients in this group died on days 7 and 10 following pneumothorax, one having been managed conservatively and the other having had their drain removed following resolution of pneumothorax. Seven patients survived to discharge with a median (IQR) length of stay of 7 (4.5–10) days.

#### Ward inpatients

14 patients developed pneumothorax during their hospital admission while breathing spontaneously on a general or respiratory ward. Of these, six were diagnosed incidentally, one was associated with marked respiratory deterioration precipitating intubation and one patient was diagnosed with tension pneumothorax. Only three patients were receiving noninvasive continuous positive airway pressure ventilation at the time of diagnosis. 11 patients required chest drains to be inserted, one of whom required right upper lobe bullectomy, as described earlier. Three (21%) of this group died and the median length of stay for the survivors was 35 days, including one patient who was later readmitted as an acute presentation with a contralateral pneumothorax.

#### Invasive ventilatory support

38 patients suffered a total of 39 pneumothoraces during invasive ventilatory support: 26 were receiving only invasive ventilation (including one patient with a tracheostomy who suffered two distinct pneumothoraces), while 12 required additional extracorporeal membrane oxygenation (ECMO).

In the ventilation cohort without ECMO, pneumothorax was diagnosed either incidentally or as a result of hypercapnia, acidosis and increasing oxygen requirement prompting investigation. At the time of diagnosis, 17 were receiving pressure-control mode ventilation, while eight were being treated with volume-control (including one patient with bilateral sequential pneumothoraces), with one case missing data on mode of ventilation. Peak pressures were not markedly elevated, with a median (IQR) of 28.0 (25.7–31.2) cmH_2_O, and 16 individuals were prone during their critical care stay, in keeping with national guidelines for the management of acute respiratory distress syndrome (ARDS) [10]. One pneumothorax was possibly related to central venous catheter insertion. Four cases had pneumomediastinum in addition to pneumothorax.

Owing to failure of invasive ventilation alone, 12 patients were also receiving ECMO at the time of their pneumothorax. In addition to ECMO, all received least-damaging lung ventilation with five having tidal volumes <100 mL. Seven of these ECMO patients were treated with chest drain insertion. Eight survived ≥28 days after development of pneumothorax.

Survival after development of pneumothorax was lower in patients receiving invasive ventilation (with or without ECMO) compared with those who were not intubated, but this difference was not statistically significant (intubated 55.8±8.4% *versus* not-intubated 77.3±8.9%; p=0.115 log-rank) (figure 3c). There was no significant difference in post-pneumothorax survival between invasive ventilation alone (49.9±10.4%) compared with those having ECMO support (68.4±13.1%) or between either subgroup of invasively ventilated patients compared with those not receiving invasive ventilation.

An apparent relationship was noted between acidosis at the time of pneumothorax and subsequent survival. Those patients receiving ventilatory support who had arterial pH <7.35 had a significantly lower survival at 28 days (35.1±11.3%) compared with those who were not acidotic (82.4±9.2%; p=0.003 log-rank). Most showed features of respiratory acidosis, although metabolic acidosis was seen in some. A significant relationship remained after inclusion of nonventilated patients for whom blood gas were available (acidotic 33.3±10.8%, not acidotic 83.3±7.6%; p=0.001) (figure 3d).

## Discussion

We have described the largest series of pneumothoraces in the context of COVID-19 that includes nonventilated patients. Although heterogeneous, these cases provide important insights into the association between pneumothorax and COVID-19. Demographically, these cases are atypical for either primary spontaneous pneumothorax, being of average height with 48% aged between 60 and 80 years, or secondary pneumothorax, with few having significant pre-existing respiratory disease or significant smoking history. Our series suggests the complication of pneumothorax is more prevalent in males (3.3:1); large series of patients with COVID-19 suggest that males are more commonly affected by severe forms the disease, which may account for this observation [1].

An observational case series cannot establish causality between COVID-19 and pneumothorax. As such, it is plausible that some cases of pneumothorax are coincidental to COVID-19. Nonetheless, given the relative frequency of this co-presentation it seems unlikely that this accounts for all, or even a majority, of our patients. The overall incidence of pneumothorax in the UK requiring emergency hospital admission is 14.1 per 100 000 per year and the proportion of the UK population having tested positive for SARS-CoV-2 up to July 3, 2020 is 0.43% (284 276 positive tests in a population of 66.8 million) [11–13]. As such, if there were no association we would anticipate throughout the entire UK there to have been 18 cases of pneumothorax diagnosed coincidentally alongside COVID-19 during the 165-day period from the UK's first confirmed case on January 22, 2020 to July 3, 2020. Despite including cases from only 16 of the UK's >150 hospital trusts, our case series is already substantially in excess of this estimate. We feel that it is reasonable to conclude that these cases are likely to represent secondary pneumothoraces as a consequence of COVID-19.

Although we are unable to provide an accurate estimate of the incidence of pneumothorax in COVID-19, we were able to obtain admissions data from the 16 centres participating in this series. This revealed that the 60 cases of pneumothorax identified in those centres were drawn from an estimated 6574 COVID-19 admissions across those sites, giving an incidence of 0.91%, which is in keeping with previously published estimates [1].

Explaining the association between these pathologies is more challenging. Radiology frequently showed typical changes of COVID-19, although in one case cavitation was thought likely to reflect pulmonary infarction. It is possible that multiple mechanisms underlie this relationship. Cyst formation in areas of airspace disease was first noted as a radiological consequence of COVID-19 soon after the initial outbreak, and has been corroborated by studies demonstrating radiological progression from areas of consolidation to bullae [14–16]. Previous reported cases have found that cyst formation is not restricted to patients receiving positive-pressure ventilation, suggesting that barotrauma alone cannot account for these findings [15]. Likewise, the fact that we report multiple cases of pneumothorax in patients who have not undergone mechanical ventilation suggests that barotrauma alone cannot explain this association. Additionally, cyst formation has been noted as a late consequence of ARDS due to SARS, the disease processes postulated including ischaemic parenchymal damage and inflammation [17].

In terms of critical-care admissions, previous analysis of intubated patients with SARS noted that tachypnoea at admission, hypoxaemia and hypercapnia all correlated with the development of pneumothorax, but there was no significant impact of ventilator pressure or volume variables, which is consistent with our data [18]. While the lower survival rate noted in intubated patients was not statistically significant, larger studies or meta-analyses may be able to further address this. We observed that acidotic patients have a significantly worse outcome, but the reason for this is unclear. Older age, another factor associated with poor outcome in our series, did not account for the apparent effect of pH, since the relationship between acidosis and poor survival persisted when only patients aged <60 years were assessed (not shown). It seems likely that acidosis in this context is an independent marker of severe disease.

Management of pneumothorax in patients with ARDS who are being invasively ventilated can represent a clinical challenge. Indeed, previous reports describe intubated patients with COVID-19 developing pneumothorax refractory to chest drain insertion which ultimately required surgery [19]. Interestingly, none of our ventilated cases progressed to require operative intervention, but we do include one case of a patient who was diagnosed on the ward with both COVID-19 and pneumothorax. In this case, air leak was persistent despite two chest drain insertions and definitive management was achieved only after bullectomy and pleurodesis, facilitating discharge from hospital. As such, for selected patients, surgical management can have positive outcomes; this is in keeping with guidance from the British Thoracic Society that persistent pneumothorax and air leak should prompt surgical referral [20].

In addition to the limitations already discussed, one issue with our work is that many of our cases were collected based on author recall and therefore we cannot be certain that case-capture was complete even within the participating institutions. Similarly, we are aware of a number of cases from across the UK which have either been published as case reports outside of this series or for whom we were unable to establish robust contact. While internet-based recruitment was a helpful tool to expand the size of our case series, our nationwide call for collaboration was restricted to only those who are familiar with Twitter: it is therefore likely that many institutions were not acquainted with our study. Conversely, we have not corrected for the possibility that some institutions who were aware of the case series had no cases to report. Consequently, we cannot accurately use our study to determine the incidence of this association within the UK. The nature of our study was by definition broadly inclusive and it is likely that the range of cases (particularly in terms of ventilated *versus* nonventilated patients) represent diverse, heterogeneous pathology rather than one distinct clinical entity.

It has previously been suggested that the development of pneumothorax during coronavirus infection is a grave prognostic marker [4, 5, 8, 9]. However, our case series does not support this, with 63.1% overall survival. Furthermore, 52% of the patients included in the series have so far survived to discharge from hospital, as compared to national figures of 41% survival to discharge for all hospital admissions (with 34% continuing to receive care) and 47% survival to discharge for those admitted to high dependency or intensive care units [21, 22]. As such, we caution against therapeutic nihilism in the context of COVID-19 pneumothorax and active treatment should be continued where clinically possible.

However, it is noteworthy that chest drain insertion for pneumothorax could reasonably be considered to be an aerosol-generating procedure, and recently SARS-CoV-2 viral RNA has been detected in pleural fluid at post-mortem [23, 24]. Therefore, it is important that clinicians be provided with appropriate personal protective equipment for aerosol-generating procedures during chest drain insertion and that consideration is given to droplet-minimising modifications to the procedure, including digital drainage systems, connection of the drainage circuit to wall suction and the use of filters to limit viral spread [25, 26]. Understanding the mechanism of the association between COVID-19 and pneumothorax is required for the development of preventative interventions.

## Supplementary material

10.1183/13993003.02697-2020.Supp1 **Please note:** supplementary material is not edited by the Editorial Office, and is uploaded as it has been supplied by the author.
Supplementary_tables ERJ-02697-2020_Supplementary_tables

## Shareable PDF

10.1183/13993003.02697-2020.Shareable1 This one-page PDF can be shared freely online.
Shareable PDF ERJ-02697-2020.Shareable
