# Supplementary material for: COVID-19 and pneumothorax: a multicentre retrospective case series
Source: Eur Respir J. 2020 Nov 19;56(5):2002697. doi: 10.1183/13993003.02697-2020 (PMC7487269; doi:10.1183/13993003.02697-2020)
Supplement: Supplementary file 1 [file ERJ-02697-2020_Supplementary_tables.pdf]

		Total (n=46)	Survived (n=31)	Died (n=15)
Hb (g/L)	Mean ± SD	107 ± 29	108 ± 28	107 ± 32
	Median [IQR]	98 [83-130]	98 [86-127]	98 [81-129]
	Range	63-173	66-163	63-173
WBC (10^9/L)	Mean ± SD	13.9 ± 6.6	13.1 ± 6.5	15.4 ± 6.6
	Median [IQR]	12.9 [8.5-18.6]	11.0 [8.3-17.2]	13.8 [9.9-20.5]
	Range	1.6-32.0	1.6-32.0	6.2-26.0
Platelets (10^9/L)	Mean ± SD	329 ± 139	346 ± 150	297 ± 107
	Median [IQR]	324 [210-423]	335 [210-444]	293 [248-385]
	Range	79-657	79-657	88-457
Neutrophils (10^9/L)	Mean ± SD	10.63 ± 5.26	9.52 ± 4.51	12.76 ± 5.91
	Median [IQR]	9.59 [6.51-13.28]	8.20 [6.30-12.51]	12.38 [8.18-16.80]
	Range	1.20-23.37	1.20-19.47	3.97-23.37
Lymphocytes (10^9/L)	Mean ± SD	1.27 ± 0.71	1.37 ± 0.77	1.06 ± 0.54
	Median [IQR]	1.10 [0.73-1.63]	1.28 [0.81-1.68]	0.84 [0.70-1.21]
	Range	0.20-3.35	0.20-3.35	0.50-2.40
Eosinophils (10^9/L)	Mean ± SD	0.25 ± 0.47	0.33 ± 0.56	0.09 ± 0.14
	Median [IQR]	0.10 [0.00-0.20]	0.11 [0.02-0.25]	0.01 [0.00-0.13]
	Range	0.00-2.60	0.00-2.60	0.00-0.50
CRP (mg/L)	Mean ± SD	134 ± 108	118 ± 105	163 ± 119
	Median [IQR]	93 [33-228]	72 [33-200]	172 [54-264]
	Range	0-360	0-333	8-360

Supplementary Table S1 Full blood counts and C-reactive protein for pneumothorax and COVID-19

Hb = haemoglobin; WBC = white blood cells; CRP = C-reactive protein; SD = standard deviation; IQR = interquartile range.

Ethnic Group	n (%)
White	34 (48%)
South Asian (Indian, Pakistani)	8 (11%)
Chinese	2 (3%)
Other Asian Background	8 (11%)
Black (African, Caribbean)	5 (7%)
Other	14 (20%)

Supplementary Table S2 Ethnicity for pneumothorax/pneumomediastinum and COVID-19
