# Supplementary material for: COVID-19 and pneumothorax: a multicentre retrospective case series
Source: Eur Respir J. 2020 Nov 19;56(5):2002697. doi: 10.1183/13993003.02697-2020 (PMC7487269; doi:10.1183/13993003.02697-2020)
Supplement: Supplementary file 2 [file ERJ-02697-2020.Shareable.pdf]

ORIGINAL ARTICLE
PULMONARY INFECTIONS

SHAREABLE PDF

COVID-19 and pneumothorax: a multicentre retrospective case series

Anthony W. Martinelli[1,2,21], Tejas Ingle[3,21], Joseph Newman[3], Iftikhar Nadeem[4], Karl Jackson[5], Nicholas D. Lane[6,7], James Melhorn[8], Helen E. Davies[8], Anthony J. Rostron[7,9], Aldrin Adeni[10], Kevin Conroy[11], Nick Woznitza[12,13], Matthew Matson[12], Simon E. Brill[14], James Murray[14], Amar Shah[14], Revati Naran[14], Samanjit S. Hare[14], Oliver Collas[14], Sarah Bigham[14], Michael Spiro[14], Margaret M. Huang[15], Beenish Iqbal[16], Sarah Trenfield[17], Stephane Ledot[17], Sujal Desai[17], Lewis Standing[18], Judith Babar[1], Razeen Mahroof[1], Ian Smith[15], Kai Lee[16], Nairi Tchrakian[19], Stephanie Uys[19], William Ricketts[19], Anant R.C. Patel[14], Avinash Aujayeb[5], Maria Kokosi[17], Alexander J.K. Wilkinson[3] and Stefan J. Marciniak[1,20]

Affiliations: [1]Addenbrooke's Hospital, Cambridge, UK. [2]CITIID, University of Cambridge, Cambridge, UK. [3]The Lister Hospital, Stevenage, UK. [4]Bedford Hospital, Bedford, UK. [5]Northumbria Specialist Emergency Care Hospital, Cramlington, UK. [6]Respiratory Medicine, The Royal Victoria Infirmary, Newcastle-upon-Tyne, UK. [7]Translational and Clinical Research Institute, Newcastle University, Newcastle upon Tyne, UK. [8]University Hospital of Wales, Cardiff, UK. [9]South Tyneside and Sunderland NHS Foundation Trust, Sunderland, UK. [10]Doncaster and Bassetlaw Teaching Hospitals NHS Foundation Trust, Doncaster, UK. [11]University Hospital of North Tees, Stockton, UK. [12]NHS Nightingale Hospital, Royal Docks, London, UK. [13]Canterbury Christ Church University, Canterbury, UK. [14]Royal Free London NHS Foundation Trust, Hampstead, London, UK. [15]Royal Papworth Hospital, Cambridge, UK. [16]King's College Hospital, London, UK. [17]Royal Brompton Hospital, London, UK. [18]Queen Elizabeth Hospital, Gateshead, UK. [19]The Royal London Hospital, London, UK. [20]CIMR, University of Cambridge, Cambridge, UK. [21]Both authors contributed equally.

Correspondence: Stefan J. Marciniak, Cambridge Institute for Medical Research (CIMR), University of Cambridge, Cambridge CB2 0XY, UK. E-mail: sjm20@cam.ac.uk

@ERSpublications
Roughly 1% of patients admitted with COVID-19 develop pneumothorax. This can occur without pre-existing lung disease or mechanical ventilation. Two-thirds of patients survive, but age >70 years and acidosis are associated with poor prognosis. https://bit.ly/2Z1mfeo

Cite this article as: Martinelli AW, Ingle T, Newman J, *et al*. COVID-19 and pneumothorax: a multicentre retrospective case series. *Eur Respir J* 2020; 56: 2002697 [https://doi.org/10.1183/13993003.02697-2020].

This single-page version can be shared freely online.

ABSTRACT
Introduction: Pneumothorax and pneumomediastinum have both been noted to complicate cases of coronavirus disease 2019 (COVID-19) requiring hospital admission. We report the largest case series yet described of patients with both these pathologies (including nonventilated patients).
Methods: Cases were collected retrospectively from UK hospitals with inclusion criteria limited to a diagnosis of COVID-19 and the presence of either pneumothorax or pneumomediastinum. Patients included in the study presented between March and June 2020. Details obtained from the medical record included demographics, radiology, laboratory investigations, clinical management and survival.
Results: 71 patients from 16 centres were included in the study, of whom 60 had pneumothoraces (six with pneumomediastinum in addition) and 11 had pneumomediastinum alone. Two of these patients had two distinct episodes of pneumothorax, occurring bilaterally in sequential fashion, bringing the total number of pneumothoraces included to 62. Clinical scenarios included patients who had presented to hospital with pneumothorax, patients who had developed pneumothorax or pneumomediastinum during

Copyright ©ERS 2020. This version is distributed under the terms of the Creative Commons Attribution Licence 4.0.

their inpatient admission with COVID-19 and patients who developed their complication while intubated and ventilated, either with or without concurrent extracorporeal membrane oxygenation. Survival at 28 days was not significantly different following pneumothorax (63.1±6.5%) or isolated pneumomediastinum (53.0±18.7%; p=0.854). The incidence of pneumothorax was higher in males. 28-day survival was not different between the sexes (males 62.5±7.7% *versus* females 68.4±10.7%; p=0.619). Patients aged ⩾70 years had a significantly lower 28-day survival than younger individuals (⩾70 years 41.7±13.5% survival *versus* <70 years 70.9±6.8% survival; p=0.018 log-rank).

Conclusion: These cases suggest that pneumothorax is a complication of COVID-19. Pneumothorax does not seem to be an independent marker of poor prognosis and we encourage continuation of active treatment where clinically possible.
